# Determination of the degree of PEGylation of protein bioconjugates using data from proton nuclear magnetic resonance spectroscopy

**DOI:** 10.1016/j.dib.2019.104037

**Published:** 2019-05-23

**Authors:** Ahlem Zaghmi, Andrea A. Greschner, Eduardo Mendez-Villuendas, Jun Yang Liu, Hendrick W. de Haan, Marc A. Gauthier

**Affiliations:** a Institut National de la Recherche Scientifique (INRS), EMT Research Center, Varennes, QC, J3X 1S2, Canada; b University of Ontario Institute of Technology, Faculty of Science, Oshawa, Ontario, L1H 7K4, Canada

## Abstract

The average number of methoxy poly(ethylene glycol) (mPEG) chains grafted to a protein – also known as the degree of PEGylation – is a fundamental parameter for characterizing a bioconjugate. The degree of PEGylation is typically determined by chromatographic or electrophoretic methods, which are subject to certain biases. This contribution describes an analytical approach alongside technical precautions for quantitatively determining the degree of PEGylation of protein bioconjugates by ^1^H NMR spectroscopy. An accompanying dataset, corresponding to the raw ^1^H NMR spectra of thirteen bioconjugates with different degrees of PEGylation and different mPEG molecular weights, is provided for the reader to become familiar with the analysis. The exemplary bioconjugate system used in this Data article is the enzyme glutamate dehydrogenase (GDH) modified with multiple copies of mPEG (0.5–20 kDa). These bioconjugates correspond to those discussed in-depth in the article “Mechanisms of activity loss for a multi-PEGylated protein by experiment and simulation” by Zaghmi et al., 2019 The described approach to calculate degree of PEGylation is quantitative, applicable to other proteins, and can be adapted to other types of polymers.

**Table:** Specifications table

Subject area	*Biochemistry*
More specific subject area	*PEGylation, Bioconjugate chemistry, Biologics, Pharmaceutical chemistry*
Type of data	*Spectra, chromatogram, tables*
How data was acquired	^1^*H NMR spectra were recorded using a Bruker Av300 spectrometer operating at* 300 MHz *for protons.* *UV–Vis absorption spectra were recorded with either a NanoDrop 2000c absorbance spectrophotometer or a Cary 60 UV–Vis spectrophotometer.* *Size-exclusion chromatography was performed using an AKTA Start fast protein liquid chromatographer equipped with a HiPrep 16/60 Sephacryl™ S200 HR column.*
Data format	*Raw and processed data*
Experimental factors	*Starting compounds were either purchased or synthesized using published procedures*[*1*]
Experimental features	*Bioconjugates were purified and the average degree of PEGylation was determined by*^1^*H NMR spectroscopy*
Data source location	*INRS, Quebec, Canada*
Data accessibility	*Data available within accompanying Supplementary Material*
Related research article	A. Zaghmi, E. Mendez-Villuendas, A.A. Greschner, J.Y. Liu, H.W. de Haan, M.A. Gauthier, Mechanisms of activity loss for a multi-PEGylated protein by experiment and simulation, Mater Today Chem. 12 (2019) 121–131, https://doi.org/10.1016/j.mtchem.2018.12.007[1].

**Table:** 

**Value of the data** • The provided dataset is useful to familiarize oneself with the methodology for determining the average degree of PEGylation of protein bioconjugates by ^1^H NMR spectroscopy. • The Data is most useful to scientists not yet familiar with the analysis of PEGylated proteins. • Precautions are provided regarding sample preparation, to acquire reliable Data for analysis. • The provided dataset and accompanying analysis can be adapted to analyze bioconjugates prepared with polymers other than mPEG. • The values obtained from the accompanying dataset highly complement those obtained by other techniques (e.g., chromatographic, electrophoretic, etc.).

## 1 Data

**Fig. 1:**
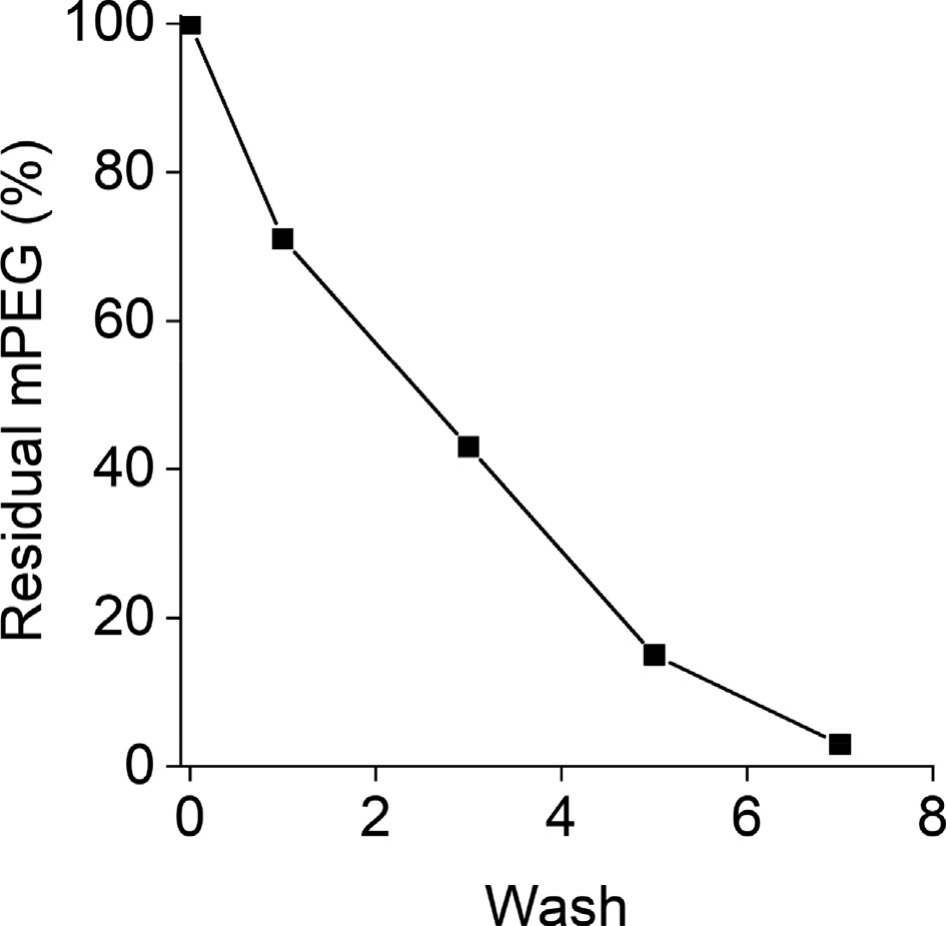
**Removal of mPEG from the crude reaction mixture of exemplary bioconjugate 12 requires multiple washes.** The integrated peak area for the main-chain methylene groups of mPEG (3.69 ppm) normalized to the intensity of the first wash. Data presented as n = 1.

**Fig. 2:**
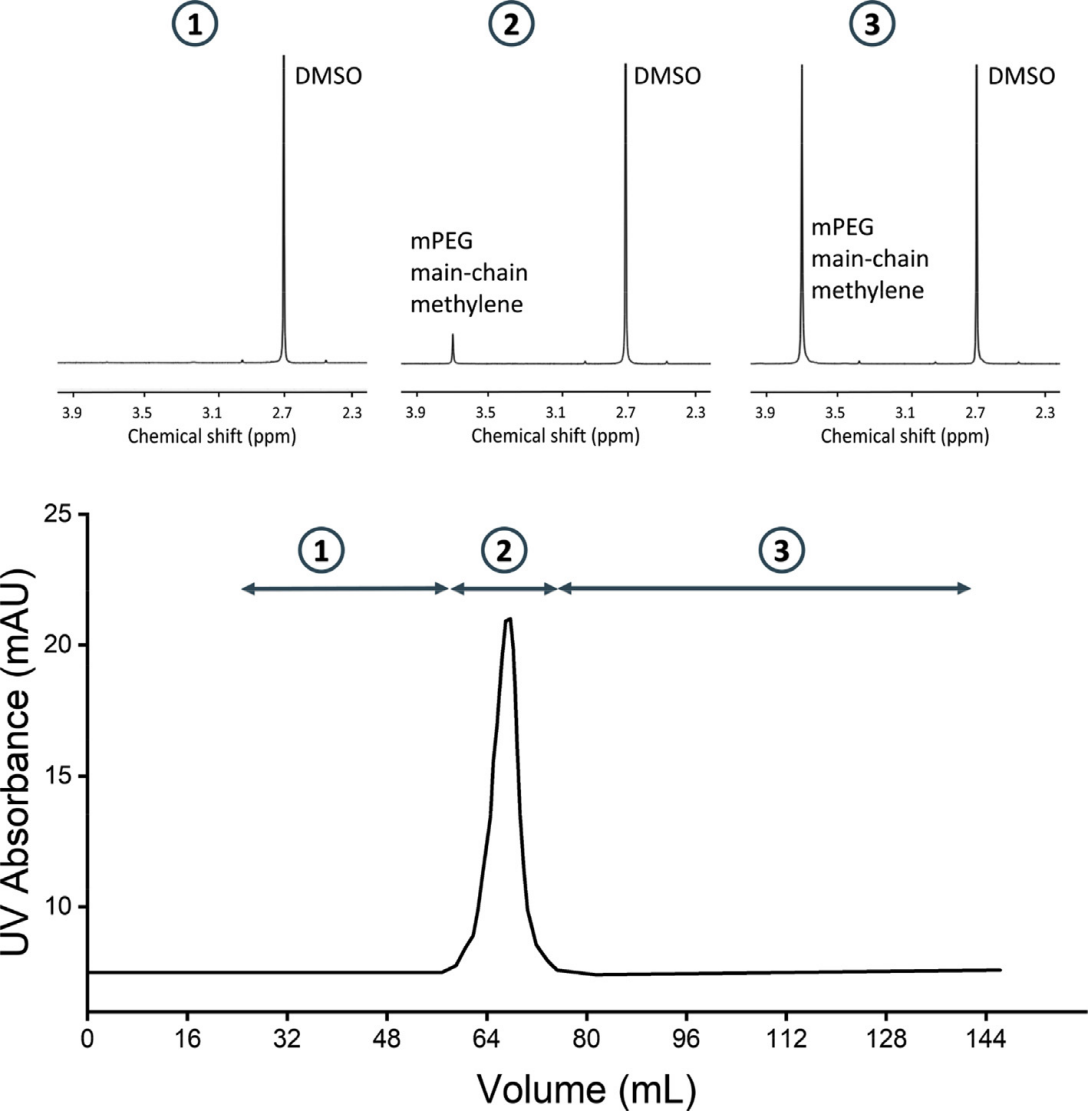
**Purification of bioconjugates by size-exclusion chromatography.** Example of a size-exclusion chromatogram of the crude bioconjugation reaction mixture of bioconjugate **12**. Analysis of pooled fractions 1, 2, and 3, corresponding to 24–56 mL (buffer), 57–76 mL (bioconjugates), and 77–144 mL (residual mPEG), respectively by ^1^H NMR spectroscopy. Note: These spectra contain peaks of DMSO (internal integration standard, *vide infra*).

**Fig. 3:**
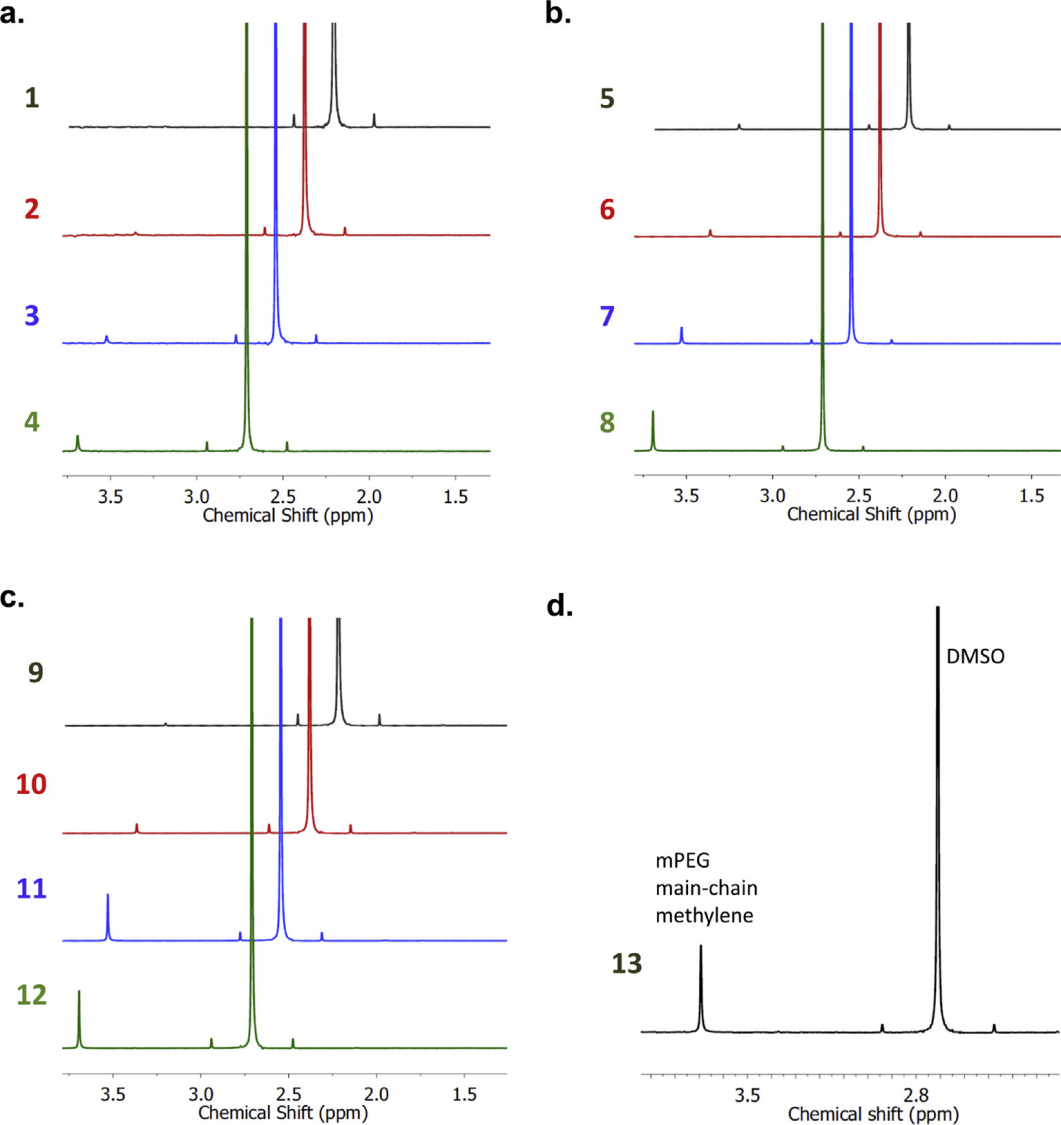
^**1**^**H NMR spectra of bioconjugates 1**–**13 in D**_**2**_**O**. Peaks corresponding to the main-chain methylene groups of mPEG and methyl groups of DMSO are identified. Bioconjugates prepared with varying mPEG molecular weights: (a) 0.5, (b) 2, (c) 5, and (d) 20 kDa. Spectra are offset in the X-axis to improve clarity.

This article provides experimental protocols and technical precautions for obtaining reliable data, suitable for determining the average degree of PEGylation of protein bioconjugates by ^1^H NMR spectroscopy. Precautions to be considered when purifying samples by centrifugal dialysis and size-exclusion chromatography yielded Fig. 1, Fig. 2. Analysis of thirteen exemplary bioconjugates by ^1^H NMR spectroscopy yielded the spectra plotted in Fig. 3. Integration of these spectra and analysis of these values yielded their average degree of PEGylation (Table 1). This article includes an accompanying dataset corresponding to the ^1^H NMR spectra of the thirteen PEGylated proteins, which can be used to become familiar with the analysis of a variety of bioconjugates with different degrees of PEGylation and different mPEG molecular weights.

**Table 1:** Data used to determine the degree of PEGylation. ‘Mn’ is the number-averaged molecular weight, ‘C’ is concentration, and ‘I’ is the integrated peak area from ^1^H NMR spectra.

Bioconjugate	M_n_ of mPEG (kDa)	mPEG: GDH ratio^a^	A_280_	C_Bioconjugate_ (μM)	I_mPEG_ (a.u.)	I_DMSO_ (a.u.)	C_mPEG_ a(μM)	Degree of PEGylation
1	0.5	1:5	1.1	3.85	0	6	0	0
2		1:30	1.0	3.25	0.01	6	6.16	2
3		1:300	1.1	3.70	0.04	6	24.6	7
4		1:1500	1.2	4.00	0.11	6	67.8	17
5	2	1:5	1.3	4.50	0.05	6	7.70	2
6		1:30	0.8	2.77	0.05	6	7.70	3
7		1:300	1.0	3.40	0.15	6	23.1	7
8		1:1500	0.9	3.23	0.31	6	47.7	15
9	5	1:5	1.0	3.37	0.058	6	3.57	1
10		1:30	0.5	1.72	0.085	6	5.24	3
11		1:300	0.5	1.67	0.275	6	16.9	10
12		1:1500	0.2	0.63	0.311	6	19.2	30
13	20	1:30	0.7	2.28	0.454	6	6.99	3

a Feed molar ratio of mPEG to GDH used to prepare the bioconjugate.

## 2 Experimental design, materials and methods

All chemicals were obtained from Sigma Aldrich (Oakville, Canada) at the highest purity available, unless otherwise specified. PEGylation of GDH using protein-reactive mPEG can be achieved by several different bioconjugate strategies [2]. For this Data article, PEGylation was achieved by reductive alkylation of bovine GDH using mPEG-propionaldehyde/mPEG-aldehyde (0.5–20 kDa; JenKem Technology (Plano, USA) and Creative PEGWorks (Durham, USA)) in the presence of sodium cyanoborohydride, as described by Zaghmi et al. [1] Other methods to prepare the PEGylated proteins could in principle be employed, as the method of protein PEGylation is not a factor that influences the calculated degree of PEGylation. Feed reaction conditions used to prepare the thirteen bioconjugates can be found in Table 1.

### 2.1 Purification of the bioconjugates

Because the described analytical method does not intrinsically involve separation of bioconjugate from starting materials (as opposed to chromatographic and electrophoretic methods), purification from residual mPEG (i.e., non-protein conjugated) is essential. Removal of non-PEGylated protein (should any remain after the reaction), may be desirable for the foreseen application of the bioconjugate. The presence or absence of residual protein will affect the degree of PEGylation measured by this method.

#### 2.1.1 Centrifugal ultrafiltration (mPEG ≤ 5 kDa)

Centrifugal ultrafiltration is the most appropriate method to eliminate residual mPEG of molecular weight below 5 kDa. However, care should be taken in the selection of the filter's Molecular Weight Cut-Off (MWCO). Most MWCOs are given in reference to globular proteins, whereas the ‘random coil’ character of mPEG results in a hydrodynamic radius substantially larger than a protein of similar molecular weight. If the MWCO is too small, little or no mPEG will pass through the filter. If the MWCO is too large, the bioconjugate itself will also pass through the filter. For 5 kDa mPEG, 15 mL Amicon ultra centrifugal filter units (or equivalent) with 100 kDa MWCO were found to give the best results. To ensure complete removal of mPEG, multiple washes should be used, and the filtrate of each wash should be analyzed for the presence of mPEG by (e.g., ^1^H NMR spectroscopy). Following the PEGylation reaction, 1 mL of the crude reaction mixture containing bioconjugate (1 mg·mL^−1^) was transferred to the top compartment of the ultrafiltration unit. Seven consecutive 15-mL washes with 100 mM sodium phosphate buffer (pH 6; total volume 105 mL) were required to remove all residual mPEG (Fig. 1). The resultant solution was lyophilized to dryness to isolate the bioconjugate. It is important to note that depending upon the feed ratio of mPEG to protein, the molecular weight of mPEG, the supplier from which the filters were purchased as well as different filters from the same supplier, these results can vary considerably. Therefore, optimization of this purification procedure is necessary for each new set of reaction/purification conditions.

#### 2.1.2 Size-exclusion chromatography (mPEG ≥ 5 kDa)

Bioconjugates prepared with higher molecular weight mPEG (e.g., ≥5 kDa) are most easily isolated from residual mPEG by size-exclusion chromatography (SEC). SEC is more appropriate for larger mPEG because its hydrodynamic radius is too large for most ultrafiltration units. Following the PEGylation reaction, the crude reaction mixture containing bioconjugate (1 mg·mL^−1^) was submitted to size-exclusion chromatography. Prior to sample injection, the HiPrep 16/60 Sephacryl™ S200 HR column was equilibrated with 0.2 column volumes of filtered and degassed sodium phosphate buffer (100 mM, pH 6) at a flow rate of 0.8 mL·min^−1^. Then, the sample (1 mL) was injected and eluted over 120 mL (1 column volume) using the same eluent and flow rate, while continuously recording absorbance at 280 nm and continuously collecting 4 mL fractions (Fig. 2). Note that mPEG propionaldehyde does not absorb at 280 nm and thus the chromatogram in Fig. 2 only shows protein-containing peaks. This is not necessarily the case for all protein-reactive mPEGs (e.g., mPEG-orthopyridyl disulfide, used to PEGylate protein thiols *via* disulfide bond formation, absorbs at 280 nm) [3]. Upon lyophilization of the fractions, white powder was evident in some tubes: those associated with the bioconjugate peak (i.e., concurrent absorbance at 280 nm) and those associated with residual mPEG (no absorbance at 280 nm). ^1^H NMR spectroscopy confirms the identity of the species present in these fractions (Fig. 2). The bioconjugate peak should be resolved from the fractions containing residual mPEG to guarantee the latter is completely removed. A second round of chromatography may be necessary if a large excess of mPEG relative to protein is used during the bioconjugation reaction. The fractions associated with the bioconjugate (57–76 mL in Fig. 2) were pooled for subsequent analysis.

### 2.2 Acquisition of data to determine the degree of PEGylation

Step 1 – Approximately ∼0.2–1 mg of the purified and lyophilized bioconjugate from either Section 1.1 or 1.2 was weighed in a 1 mL Eppendorf tube.

Step 2 – Exactly 500 μL of D_2_O was added to the Eppendorf and quantitatively transferred to a UV-transparent cuvette. Absorbance at 280 nm was recorded using a standard UV–Vis spectrophotometer. Alternatively, a small volume (ca. 2 μL) of the bioconjugate solution was analyzed in the Nanodrop spectrometer.

Step 3 – The concentration of bioconjugate in solution was calculated using the extinction coefficient of the protein (292,920 cm^−1^·M^−1^ for hexameric GDH). The concentration of protein was considered equivalent to the concentration of bioconjugate (C_Protein_ = C_Bioconjugate_).

Step 4 – The bioconjugate solution was transferred quantitatively into an NMR tube and precisely 1–5 μL of dimethyl sulfoxide (DMSO) was added quantitatively, to be used as an internal integration standard. Note that DMSO is a convenient internal standard because it can be removed alongside water by lyophilization, for sample recovery.

Step 5 – A standard ^1^H NMR spectrum was recorded of this solution. The ^1^H NMR spectra of bioconjugates **1**–**13** are presented in Fig. 3, and show two major peaks of interest: a singlet at 3.69 ppm for the main-chain ethylene groups of mPEG, and a singlet at 2.71 ppm for the methyl groups of DMSO. Peaks characteristic of the protein component of the bioconjugate are not generally observed.

Step 6 – The concentration of mPEG in solution (C_mPEG_) is determined by integration of the peak of mPEG (I_mPEG_; main-chain ethylene group at 3.69 ppm) relative to the integration standard DMSO (I_DMSO_; methyl group at 2.71 ppm) *via*:

(1) $$C_{mPEG}=\frac{6}{I_{DMSO}}\times \frac{I_{DMSO}}{4\times DP_{mPEG}}\times C_{DMSO}$$

where DP_mPEG_ is the degree of polymerization of the mPEG (113 in the case of 5 kDa mPEG), ‘6’ is the number of protons on a molecule of DMSO, ‘4’ is the number of protons per repeat unit in mPEG, and C_DMSO_ is 0.084–0.28 M based the sample preparation conditions described above.

Step 7 – The average degree of PEGylation is given by:

(2) $$Degree\;of\;PEGylation=\frac{C_{mPEG}}{C_{Bioconjugate}}$$

Values obtained from the spectra in Fig. 3 for bioconjugates **1**–**13** are found in Table 1. The accompanying dataset contains the raw data for the ^1^H NMR spectra of the thirteen PEGylated proteins, which can be used to become familiar with the analysis of a variety of bioconjugates with different degrees of PEGylation and different mPEG molecular weights.
